# Irisin ameliorates neuroinflammation and neuronal apoptosis through integrin αVβ5/AMPK signaling pathway after intracerebral hemorrhage in mice

**DOI:** 10.1186/s12974-022-02438-6

**Published:** 2022-04-07

**Authors:** Yao Wang, Mi Tian, Jiaying Tan, Xu Pei, Chaocheng Lu, Yuewen Xin, Shuixiang Deng, Feng Zhao, Yanqin Gao, Ye Gong

**Affiliations:** Department of Critical Care Medicine and Neurosurgery of Huashan Hospital, State Key Laboratory of Medical Neurobiology and MOE Frontiers Center for Brain Science, Institutes of Brain Science, Fudan University, Shanghai, China

**Keywords:** Integrin αVβ5, Intracerebral hemorrhage, Irisin, AMPK, Neuroinflammation

## Abstract

**Background:**

Neuroinflammation is a crucial factor in the development of secondary brain injury after intracerebral hemorrhage (ICH). Irisin is a newly identified myokine that confers strong neuroprotective effects in experimental ischemic stroke. However, whether this myokine can exert neuroprotection effects after ICH remains unknown. This study aimed to investigate the impact of irisin treatment on neuroinflammation and neuronal apoptosis and the underlying mechanism involving integrin αVβ5/AMPK pathway after ICH.

**Methods:**

Two hundred and eighty-five adult (8-week-old) male C57BL/6 mice were randomly assigned to sham and ICH surgery groups. ICH was induced via intrastriatal injection of autologous blood. Irisin was administered intranasally at 30 min after ICH. To elucidate the underlying mechanism, cilengitide (a selective integrin αVβ5 inhibitor) and dorsomorphin (a selective phosphorylated AMPK inhibitor) were administered before irisin treatment. The short- and long-term neurobehavior tests, brain edema, quantitative-PCR, western blotting, Fluoro-Jade C, TUNEL, and immunofluorescence staining were performed to assess the neurofunctional outcome at the level of molecular, cell, histology, and function.

**Results:**

Endogenous irisin and its receptor, integrin αVβ5, were increased, peaked at 24 h after ICH. irisin post-treatment improved both short- and long-term neurological functions, reduced brain edema after ICH. Interestingly, integrin αVβ5 was mainly located in the microglia after ICH, and irisin post-treatment inhibited microglia/macrophage pro-inflammatory polarization and promoted anti-inflammatory polarization. Moreover, irisin treatment inhibited neutrophil infiltration and suppressed neuronal apoptotic cell death in perihematomal areas after ICH. Mechanistically, irisin post-treatment significantly increased the expression of integrin αVβ5, p-AMPK and Bcl-2, and decreased the expression of IL-1β, TNF-α, MPO, and Bax following ICH. The neuroprotective effects of irisin were abolished by both integrin αVβ5 inhibitor cilengitide and AMPK inhibitor dorsomorphin.

**Conclusions:**

This study demonstrated that irisin post-treatment ameliorated neurological deficits, reduced brain edema, and ameliorated neuroinflammation and neuronal apoptosis, at least in part, through the integrin αVβ5/AMPK signaling pathway after ICH. Thus, irisin post-treatment may provide a promising therapeutic approach for the early management of ICH.

**Supplementary Information:**

The online version contains supplementary material available at 10.1186/s12974-022-02438-6.

## Introduction

Intracerebral hemorrhage (ICH) is a severe fatal subtype of stroke accounting for approximately 10 to 15% of all stroke patients, with high rates of mortality and morbidity [1, 2]. The formation of a huge hematoma and mechanical compression to surrounding brain tissues after sudden rupture of cerebral blood vessels results in intracranial hypertension and neurological deterioration, which is regarded as the primary brain injury [3]. However, surgical intervention of the hematoma in ICH patients used in clinical practice rarely improves the neurological prognosis of patients [4]. Secondary brain injury (SBI) refers to a series of pathological processes triggered by red blood cell debris and degradation products, such as inflammatory response, mitochondrial dysfunction, oxidative stress, neuronal apoptosis, and blood–brain barrier (BBB) disruption [5–8]. In recent decades, novel therapeutic targets have been focused on exploring the mechanisms underlying ICH-induced SBI.

Neuroinflammation is an immediate host defense response after the presence of blood components detected within the parenchyma, which plays a pivotal role in SBI after ICH [9]. The inflammatory mechanisms consist of activation of microglia and infiltration of various peripheral immune cells to the perihematomal region [6]. This subsequently leads to the release of proinflammatory cytokines (e.g., interleukin (IL)-1β and tumor necrosis factor (TNF)-α), chemokines, free radicals, and other potentially toxic chemicals, which further promotes a continued cycle of the inflammatory cascade, contributes to perihematomal edema, BBB disruption and cell death [10–12]. Neuronal apoptosis could cause neuronal loss at the periphery of the clot, which ultimately aggravates inflammatory injury and tissue damage [13]. Therefore, a therapeutic strategy targeting anti-inflammation and anti-apoptosis may be crucial for improving neurological outcomes after ICH.

As a newly myokine first identified in 2012, irisin is formed by proteolytic cleavage of the N-terminal fragment of fibronectin type III domain-containing protein 5 (FNDC5), a transmembrane precursor protein mainly expressed in muscle. Irisin is a polypeptide consisting of 112 amino acids, and it was initially described as a myokine released into the circulation on physical exercise mainly by muscle and a tiny portion by adipose tissue, which is capable of stimulating adipocyte browning and thermogenesis in humans and mice [14, 15]. Recently, the existence of irisin has been verified in various brain regions and cellular groups, such as astrocytes in hippocampus, neuron in cerebrum and even in cerebrospinal fluid [16–18]. Irisin is shown to play a neuroprotective role in brain disorders, such as Alzheimer’s disease (AD), and acute brain injuries, such as ischemic stroke and traumatic brain injury [19–21]. Moreover, irisin has been demonstrated to show pivotal roles in attenuating inflammation, reducing neuronal apoptosis, and alleviating oxidative stress [22]. However, the possible role of irisin on neuroinflammation and neuronal apoptosis has not been investigated in the setting of ICH. Recently, integrin αVβ5 has been identified as a functioning receptor for irisin in both osteocyte and intestinal epithelial cells, and both biochemical and biophysical studies identified interactions between irisin and integrin αVβ5 [23, 24]. Intriguingly, it’s reported that integrin αVβ5 is highly expressed in the microglia/macrophage [25]. Whether irisin could alleviate neuroinflammation through binding to integrin αVβ5 expressed in the microglia/macrophage has not been elucidated yet.

Adenosine 5’monophosphate-activated protein kinase (AMPK) as a cellular energy sensor, is a key molecule in the regulation of bioenergy metabolism. Potent evidence revealed that phosphorylation of AMPK at the site of Thr172 could mitigate cell apoptosis and alleviate neuroinflammation in various pathophysiological processes [26–29]. AMPK activation (phosphorylation of α subunit at thr172) is suggested to alleviate inflammation and promote microglial/macrophage polarization to the M2-phenotype [30, 31]. Many studies show that irisin can promote AMPK activation to regulate energy metabolism [32]. Besides, AMPK signaling is recognized as a downstream target pathway after irisin binding with integrin αVβ5 receptor [33]. However, whether integrin αVβ5/AMPK signaling is associated with irisin-mediated neuroinflammation and neuronal apoptosis requires further elucidation.

In the current study, we hypothesized that irisin treatment could ameliorate neurological deficits and alleviate neuroinflammation and neuronal apoptosis through integrin αVβ5/AMPK signaling pathway after ICH in mice.

## Methods

### Animals

Male C57BL/6 mice, 8–10 weeks (25 ~ 30 g), were purchased from Shanghai JieSiJie Laboratory Animal Co., Ltd. Mice were housed in a 12-h light/dark cycle at a constant temperature and humidity controlled room for a minimum of 3 days before surgery, with free access to food and water. All animal experiments in the study were approved by the Animal Care and Use Committee of Shanghai Medical College, Fudan University.

### Experimental design

In the present study, all mice were randomly assigned to the following experiments. The experimental design was shown in Part 1 of the Additional file 1: Fig. S1. The summary of experimental groups, animal numbers, and mortality rate in the study was listed in Part 2 of the Additional file 1: Table S1.

### ICH model

ICH surgery was induced by stereotactic-guided injection of autologous whole blood into the right basal ganglia as previously described [34]. Briefly, mice were anesthetized with an isoflurane–oxygen mixture during the surgical procedure, and the body temperature was maintained at 37.0 ± 0.5 °C using a heating blanket. Mice were positioned prone on the stereotactic head frame (Kopf Instruments, Tujunga, CA, USA). An artificial tears ointment was applied to keep the eyes moist during surgery. Arterial blood was collected in a nonheparinized capillary tube and transferred immediately into a 27-gauge needle on a 250 μL Hamiton syringe. A coronal incision was performed to expose the cranium until the bregma was clearly visible. After that, a 1-mm cranial burr hole was drilled in the skull, and the Hamilton syringe was inserted into the right basal ganglia following the stereotactic guide (coordinates: 0.2 mm posterior, 2.2 mm lateral to the bregma). Following this, a total volume of 30 μL autologous blood (initial 5 μL at depth of 3.0 mm below dura and followed by 25 μL at the depth of 3.5 mm below dura 5 min later) was infused using a microinfusion pump (Stoelting, Harvard Apparatus, Holliston, MA) at a rate of 2 μL/min. To prevent possible leakage due to blood backflow, the needle was left in place for an additional 10 min after the completion of a 30 μL injection and slowly withdrawn at a rate of 1 mm/min. The burr hole was then sealed with sterilized medical bone wax and the incision of the scalp was sutured. Mice were then allowed to recover fully on a heating pad at 37 °C, and the neurological deficits were closely observed. The sham surgery was performed following the same procedure without blood injection.

### Drug administration

Recombinant irisin (#100-65, Peprotech, USA) was dissolved in phosphate-buffered saline (PBS). Three different doses of irisin were tested (80 μg/kg, 250 μg/kg, and 750 μg/kg) and were administered intranasally at 30 min after ICH induction [35]. Cilengitide trifluoroacetate (10 mg/kg, Selleck, USA), a selective inhibitor of αVβ3 and αVβ5 integrins, was dissolved in DMSO and administered intraperitoneally at 2 h before ICH induction [36]. Dorsomorphin (5 μg/mouse, Sigma, MO), a selective AMPK inhibitor, was dissolved in DMSO and administered intracerebroventricularly (i.c.v.) 30 min before ICH injury [37].

### Intracerebroventricular injection

Intracerebroventricular administration was performed as previously described [38]. Briefly, a 1-mm cranial burr hole was drilled at the following coordinates relative to bregma: 0.22 mm posterior, 1.0 mm lateral). A 26-gauge needle of a 10 μL Hamilton syringe was inserted into the left lateral ventricle through the cranial burr hole at the depth of 2.25 mm deep under dura. A microinfusion pump was used for intracerebroventricular injection at a rate of 0.667 μL/min. The needle was left in place for an additional 5 min at the end of infusion and then removed slowly over a 3-min period. The burr hole was immediately sealed with sterilized medical bone wax.

### Short-term neurobehavioral assessment

Short-term neurofunctional behavior was assessed with modified Garcia score test, forelimb placement test, and corner turn test at 24 h and 72 h post-ICH by an independent researcher who was blinded to the information of experimental groups, as previously described [39]. The modified Garcia score was assessed by a 21-point score system with seven individual tests including spontaneous activity, axial sensation, vibrissae proprioception, limb symmetry, lateral turning, forelimb walking, and climbing. Each subtest was scored from 0 to 3, and the total score was generated by adding up the sum of seven subtest scores. For the forelimb placement test, the placement of the left forelimb on the countertop when the vibrissa was stimulated was recorded. The percentage of the left forelimb placement out of ten consecutive vibrissae stimulations was calculated. The corner turn test was performed utilizing the device which consisted of two boards forming a 30° angle vertically on the platform. The mice were allowed to advance into a 30° angel corner and exit by turning either to the left or the right. Choice of turning was recorded for a total of ten trials, and the result was the percent of left turns in 10 trials.

### Long-term neurobehavioral assessment

Rotarod test was performed to evaluate the sensorimotor function, coordination, and balance on days 7, 14, and 21 post-ICH with 47,650 Mouse Rota-Rod (UGO BASILE). The mice to be tested were placed in each lane on the rotating cylinder at a speed of 5 r/min and accelerated to 40 r/min within 300 s and went on rotating at the constant speed for 200 s (total 500 s). The falling latency was recorded, which was defined as the time duration when a mouse stabilizes itself on the rotating cylinder without falling. Three trials were performed and the mean time of falling latency was recorded. Morris water maze was carried out to evaluate spatial learning and memory abilities on days 21–26 after ICH, as previously reported [39]. In the learning phase of the test, the mouse was placed into the pool from one of the three quadrants without the platform and then allowed to swim for up to 60 s to escape to the hidden platform. The time that the animal spent to find the platform (escape latency) was recorded for each trial as “spatial learning”. At the end of each trial, the mouse was allowed to remain on the platform or placed on the platform (if the mouse could not find the platform within 60 s) for 10 s with prominent spatial cues displayed around the room. Mice were pre-trained for 3 consecutive days before ICH induction (3 trials on each day). After the injury, 3 trials were conducted on each testing day for 5 consecutive days (21–25 days after ICH). In the memory phase of the test at 26 days after ICH, a single, 60-s probe trial was performed with the platform removed. The time the mouse spent swimming in the goal quadrant, where the platform was previously located was recorded as “spatial memory” and expressed as a percentage of the total testing time of 60 s.

### Brain water content measurement

Brain water content (BWC) was measured through the wet/dry method as previously reported [40]. Briefly, mice were euthanized by decapitation under deep anesthesia at 24 and 72 h post-ICH. Brains were removed immediately and then divided into five parts: ipsilateral and contralateral cortex, ipsilateral and contralateral basal ganglia, and cerebellum. Each brain section was measured immediately on an analytical microbalance to obtain the wet weight (WW) and then dried for 24 h at 100 °C to obtain the dry weight (DW). Brain water content was calculated through the following formula: brain water content (%) = [(WW − DW)/WW] × 100%.

### Enzyme-linked immunosorbent assay (ELISA)

Plasma levels of irisin were measured at 6 h and 24 h after injury following the manufacturer’s instructions of a commercial ELISA kit (Phoenix Pharmaceutical, Burlingame, CA) with a 1:2 dilution of each plasma sample (50 μL). Intra- and inter-assay variances were < 4–6% and < 8–10%, respectively, and the range of detectable concentrations was 0.066–1024 ng/ml.

### Quantitative real-time polymerase chain reaction (q-PCR)

Total RNA was extracted from brain tissue 24 h after ICH around the hemorrhage using Trizol (Qiagen, Hilden, Germany) according to the manufacturer’s protocol, afterwards, RNA was reverse transcribed into cDNA using Superscript III First-Strand Synthesis SuperMix (Invitrogen, Carlsbad, CA, USA). Quantitative real-time polymerase chain reaction (q-PCR) was performed using synthetic primers listed in Part 3 of the Additional file 1 and SYBR GREEN FAST mastermix (Qiagen). Data collection was performed on the RT-PCR System (Bio-Rad, Hercules, CA, USA). GAPDH was used as an internal control. The relative quantitation value for each gene was performed using the comparative cycle threshold method [41].

### Western blot analysis

Mice were transcardially perfused with cold PBS under deep anesthesia at 24 h post-ICH. Brain samples were stored at -80 freezers after quickly extracted and snap-frozen in liquid nitrogen. Western blotting was performed as previously described [42]. Briefly, after brain samples were homogenized using RIPA lysis buffer (Santa Cruz Biotechnology, Santa Cruz, CA, USA) and centrifuged at 4 °C for 30 min at 14,000 rpm, equal amounts of protein were loaded on an SDS–PAGE gel and run using electrophoresis and then transferred to a nitrocellulose membrane. Equal amounts of protein were loaded on an SDS–PAGE gel and run using electrophoresis, and then transferred to a nitrocellulose membrane. The membrane was blocked and then incubated overnight at 4 °C with the following primary antibodies: rabbit anti-irisin (1:1000, ab174833, Abcam, USA); rabbit integrin αV (1:1000, ab179475, Abcam, USA); rabbit anti-integrin β5 (1:1000, #3629, Cell Signaling Technology, Inc., MA, USA); anti-AMPKα (1:1000, #5831, Cell Signaling Technology, Inc., MA, USA); anti-p-AMPKα (1:1000, #2535, Cell Signaling Technology, Inc., MA, USA); rabbit anti-IL-1β (1:1000, #12242, Cell Signaling Technology, Inc., MA, USA); rabbit anti-Iba-1 (1:1000, ab178846, Abcam, USA); rabbit anti-MPO (1:1000, ab208670, Abcam, USA); rabbit anti-TNF-α (1:1000, #11948, Cell Signaling Technology, Inc., MA, USA); rabbit anti-Bcl-2 (1:2000, ab182858, Abcam, USA), rabbit anti-Bax (1:4000, ab182733, Abcam, USA). The membranes were incubated with mouse anti-β-actin (1:2000, sc-47778, Santa Cruz, USA) as a loading control. Appropriate secondary antibodies (1:3000, Santa Cruz; 1:5000, Abcam) were selected to incubate with the membrane for 2 h at room temperature. The membranes were then incubated with horseradish peroxidase-conjugated goat anti-rabbit IgG or goat anti-mouse IgG (Proteintech) secondary antibodies. The bands were probed with an ECL Plus chemiluminescence reagent Kit (Amersham Biosciences, Arlington Heights, PA, USA) and visualized with the imaging system (Versa Doc, model 4000, Bio-Rad, Hercules, CA, USA). The relative density of the protein immunoblot images was analyzed by ImageJ software (Image J 1.5, NIH, USA).

### Immunofluorescence staining

After being anesthetized deeply at 24 h and 72 h after ICH, mice were transcardially perfused with ice-cold PBS and 4% paraformaldehyde. Brains were then removed and immersed in 4% paraformaldehyde, 20% sucrose, and 30% sucrose successively to complete fixation and dehydration. Coronal sections (25-μm thick) were sliced using the freezing microtome (HM525NX, ThermoFisher), and stored in tissue stock solution. After being washed in PBS and PBS + 0.3% Triton, coronal sections were incubated in PBS + 1% Triton to break the cell membrane and blocked with 10% goat/donkey serum for 1 h. Coronal sections were incubated with primary antibodies overnight at 4 °C. After being washed in PBS three times with 10 min intervals, the sections were incubated with secondary antibodies conjugated with Alexa Fluor-488/594/647 for 2 h at room temperature. Nuclear staining was performed with 4′,6-diamidino-2-phenylindole (DAPI). Sections were then observed and imaged under a Nikon microscope (Nikon).

### TUNEL staining

For quantification of neuronal apoptosis at 24 h after ICH, double staining of neuron marker NeuN (red) and TUNEL (green) was conducted using in situ Apoptosis Detection Kit (Roche, Indianapolis, IN, USA) according to the manufacturer’s instructions [43]. In the peri-hematoma area, TUNEL-positive neurons were counted manually and the numbers of random six sections per brain slice over a microscopic field of 200× magnifications using Image J software (Image J 1.5, NIH, USA) were averaged. Data were expressed as the ratio of TUNEL-positive neurons (%).

### FJC staining

Degenerating neurons was evaluated by FJC staining using a modified FJC Ready-to-Dilute Staining Kit (Millipore, Billerica, MA, USA) at 24 h post-ICH as previously reported [44]. According to the manufacturer’s instructions, slides were washed in PBS incubated with the FJC working solution for 20 min and then visualized using a fluorescence microscope (Leica Microsystems) in a blinded manner. FJC-positive neurons were manually counted in the peri-hematoma regions of six parts per brain at ×200 magnification using ImageJ software (Image J 1.5, NIH, USA). The data were averaged and expressed as positive cells/mm^2^.

### Statistical analysis

All data are expressed as mean ± standard deviation (SD). Data were normally distributed as tested using the D’Agostino and Pearson omnibus normality test (*p* > 0.05). For comparisons between 2 groups, the Student’s *t* test was used for comparisons of variables with normal distribution from independent samples. Multiple comparisons were statistically analyzed using one-way analysis of variance (ANOVA) followed by Tukey post hoc multiple comparison analysis. Two-way ANOVA followed by Tukey post hoc test was used to compare the changes according to the different levels of multiple categorical variables (brain water content, long-term neurological function). Data analyses were conducted using Prism 9 (GraphPad Software, CA, USA). All statistical tests were two-sided, and a *p* < 0.05 was considered statistically significant.

## Results

### Animal mortality and exclusion

A total of 285 male C57BL/6 mice were used for the study, among 203 animals were assigned to ICH induction and a total of 3 ICH mice were excluded, because they did not show neurological deficits after surgery. (After euthanizing these mice, we found that there was no hematoma in their brains.) None of the sham group mice died. The total mortality of ICH mice was 4.93% (10/203) in this study. There were no significant differences in the mortality rate among all ICH experimental groups. (Part 2 of the Additional file 1: Table S1).

### Time course and cellular expression of irisin and integrin αVβ5 after ICH

**Fig. 1:**
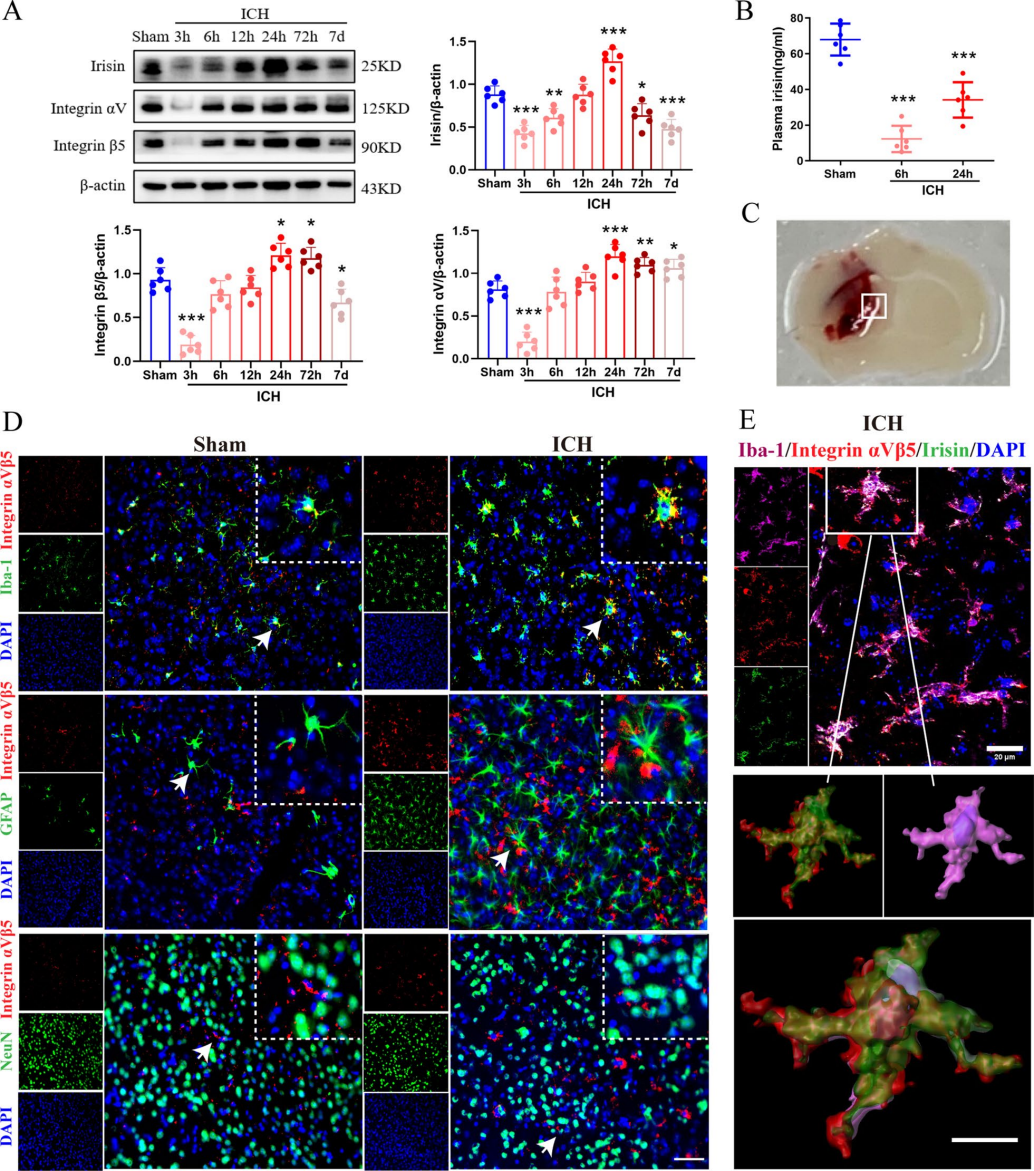
Expression profile of irisin and integrin αVβ5 after ICH. **A** Representative western blot bands of time course and quantitative analyses of irisin and integrin αVβ5 expression in the ipsilateral hemisphere after ICH. *p < 0.05, **p < 0.01, ***p < 0.001 vs. sham group. Error bars are represented as mean ± SD. *n* = 6 per group. **B** Comparison of plasma irisin levels between Sham and ICH mice at 6 h and 24 h after ICH surgery. *p < 0.05, **p < 0.01, ***p < 0.001 vs. sham group, *n* = 6 per group. **C** Schematic illustration of brain tissue showing the area in the perihematomal region (indicated by white box) from where the images were taken for immunofluorescence staining. **D** Representative images of colocalization of integrin αVβ5 (red) with microglia/macrophage (Iba-1, green), astrocytes (GFAP, green) and neurons (NeuN, green) in the sham group and the perihematomal area of ICH (24 h) group. Nuclei were stained with DAPI (blue). Scale bar = 50 μm, *n* = 2/group. **E** Triple-label immunofluorescence confocal microscopy for microglia/macrophage marker Iba-1 (magenta), integrin αVβ5 (red), and irisin (green) in the perihematomal region at 24 h after ICH. Rectangle: cell enlarged and three-dimensional (3D)-rendered by Imaris 9.2 (Bitplane, Switzerland) in the down panel. Nuclei were stained with DAPI (blue). Scale bar of the 3D-rendered cell = 10 μm

To characterize the expression profile of irisin and its receptor integrin αVβ5, western blot analysis, ELISA, and immunofluorescence staining were conducted. The endogenous expression of irisin and integrin αVβ5 was assessed by western blot analysis at 0 (sham), 3, 6, 12, 24, 72 h and 7 days in the ipsilateral/right cerebral hemispheres after ICH. Compared to the sham group, irisin expression significantly decreased at 3 h and 6 h and then increased at 12 h, which peaked at 24 h after ICH (*p* < 0.001, Fig. 1A). The expression of integrin αV and integrin β5 showed a similar trend after ICH (Fig. 1A). ELISA was performed to assess the plasma levels of irisin at 6 h and 24 h after ICH. The plasma irisin concentration in the sham group was 67.96 ng/ml on average (Fig. 1B), and there was a notable decrease in plasma irisin levels detected at 6 h (12.28 ng/ml) and 24 h (34.12 ng/ml) after ICH (*p* < 0.001, Fig. 1B). Double immunofluorescence staining revealed that integrin αVβ5 was mainly expressed in the microglia/macrophages (Iba-1^+^) in the peri-hematoma tissue at 24 h after ICH, whereas integrin αVβ5 was not co-localized with astrocytes and neurons in the mouse brain (Fig. 1C, D). Meanwhile, there were more integrin αVβ5-positive microglia visualized within the perihematomal region at 24 h post-ICH when compared with the sham group (Fig. 1C, D). In addition, irisin was localized with the microglia/macrophages both in the sham and ICH groups, and irisin expression in the microglia/macrophages was upregulated after ICH (Additional file 1: Fig. S2). To further investigate the distribution characteristics of irisin and integrin αVβ5 in the perihematomal region at 24 h after ICH, triple immunofluorescence confocal microscopy of Iba-1 (magenta), integrin αVβ5 (red), and irisin (green) were performed and visualized by confocal image stacks (scale bar = 20 μm). The coincidence of white fluorescence with magenta, red and green fluorescence indicated an observable co-localization of irisin and integrin αVβ5 in the microglia/macrophage cells (Fig. 1E, up panel). Iba-1^+^/ integrin αVβ5^+^/irisin^+^ immunolabeled cell in the rectangle of the confocal image stacks was enlarged and reconstructed to 3D isosurfaces by Imaris, which provided better visualization of co-localization of irisin and integrin αVβ5 in the microglia/macrophage cells (Fig. 1E, down the panel, scale bar = 10 μm).

### Irisin treatment attenuated neurobehavioral deficits and reduced brain edema at 24 and 72 h after ICH

**Fig. 2:**
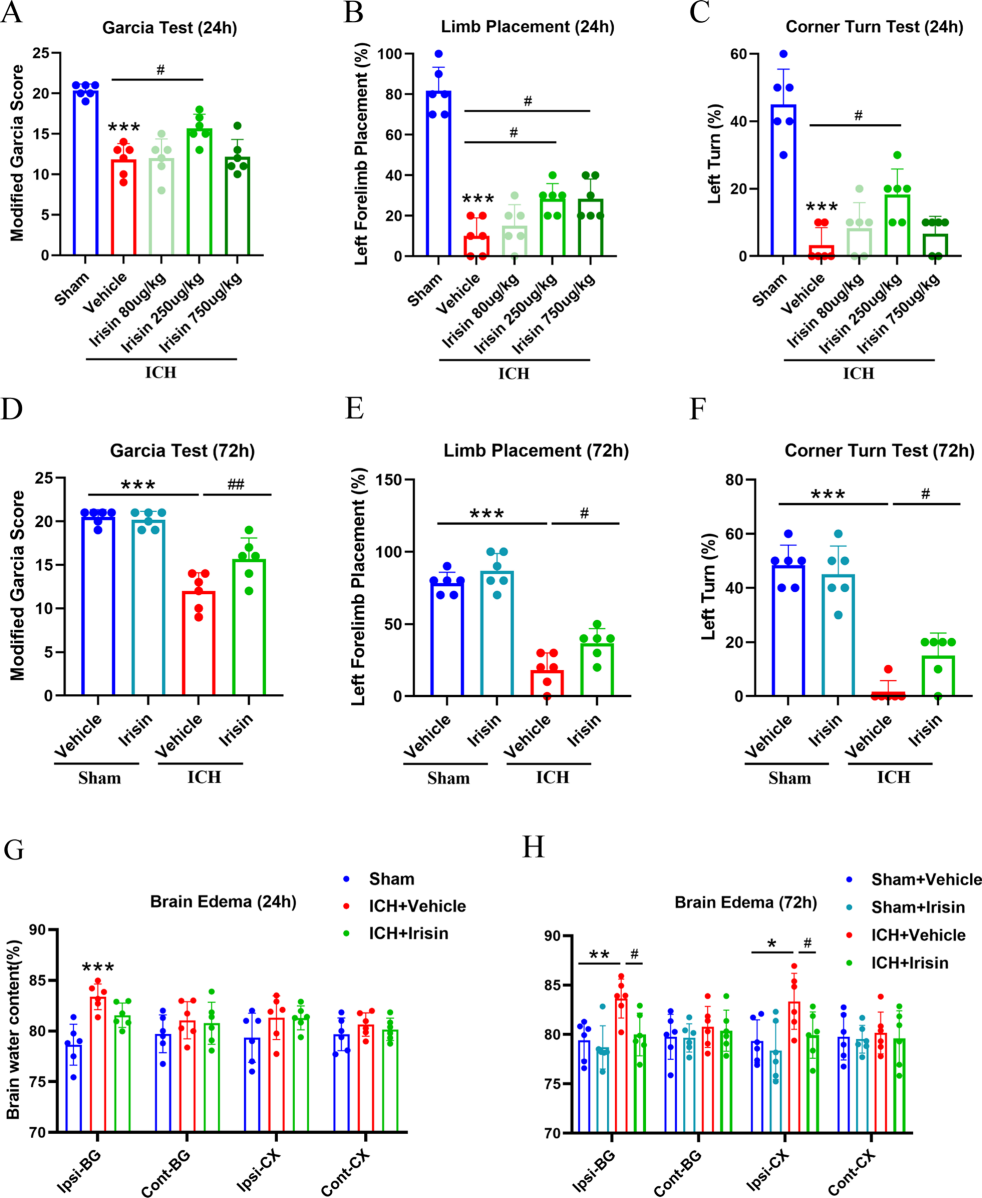
Effects of different doses of irisin on neurobehavior tests, brain water content (BWC) at 24 h and 72 h post-ICH. **A** Modified Garcia test, **B** forelimb placement test, **C** corner turn test, and G. BWC at 24 h post-ICH. **D** Modified Garcia test, **E** forelimb placement test, **F** corner turn test, and **H** BWC at 72 h after ICH. *p < 0.05, **p < 0.01, ***p < 0.001 vs. sham or sham + vehicle group; #*p* < 0.05, ##*p* < 0.01 vs. ICH + vehicle group. Error bars are represented as mean ± SD. One-way ANOVA, Tukey post hoc test, *n* = 6 per group. Ipsi-BG, ipsilateral basal ganglia; Cont-BG, contralateral basal ganglia; Ipsi-CX, ipsilateral cortex; Cont-CX, contralateral cortex

Three different dosages of irisin were used to choose the optimal dosage in attenuating ICH-induced brain injury. Significant neurological deficits were observed in ICH groups at 24 h when compared with the sham group as assessed by the modified Garcia test (*p* < 0.001, Fig. 2A), forelimb placement test (*p* < 0.001, Fig. 2B), and corner turn test (*p* < 0.001, Fig. 2C). Administration of irisin (250 μg/kg) significantly improved neurological outcomes (*p* < 0.05, Fig. 2A–C) at 24 h post-ICH when compared with ICH + vehicle group. The brain water content in the ipsilateral basal ganglia was significantly increased in the ICH groups when compared with the sham group at 24 h after ICH (*p* < 0.001, Fig. 2G), whereas no significant differences were observed between irisin administration (250 μg/kg) and ICH + vehicle group at 24 h post-ICH. To further verify the protective effect of irisin (250 μg/kg), neurobehavioral tests and brain water content were also performed at 72 h post-ICH. No obvious difference was observed between irisin and vehicle-treated (PBS) administration in sham mice. Consistently, irisin (250 μg/kg) treatment significantly improved neurological functions (*p* < 0.01, Fig. 2D, F) and alleviated brain water content in the ipsilateral basal ganglia and ipsilateral cortex (*p* < 0.05, Fig. 2H) when compared with ICH + vehicle group at 72 h post-ICH. Therefore, a middle dosage of irisin (250 μg/kg) was selected for long-term and mechanistic studies. To further compare the time-course of irisin level in the perihematomal area between ICH + vehicle and ICH + irisin (250 μg/kg) groups, western blot analysis was conducted. Compared with the ICH + vehicle group, the irisin post-treatment group maintained a high and stable level of irisin, characterized by elevating the level of irisin, while it is decreased significantly for 7 days after ICH (Additional file 1: Fig. S3).

### Irisin treatment improved long-term neurobehavioral outcomes after ICH

**Fig. 3:**
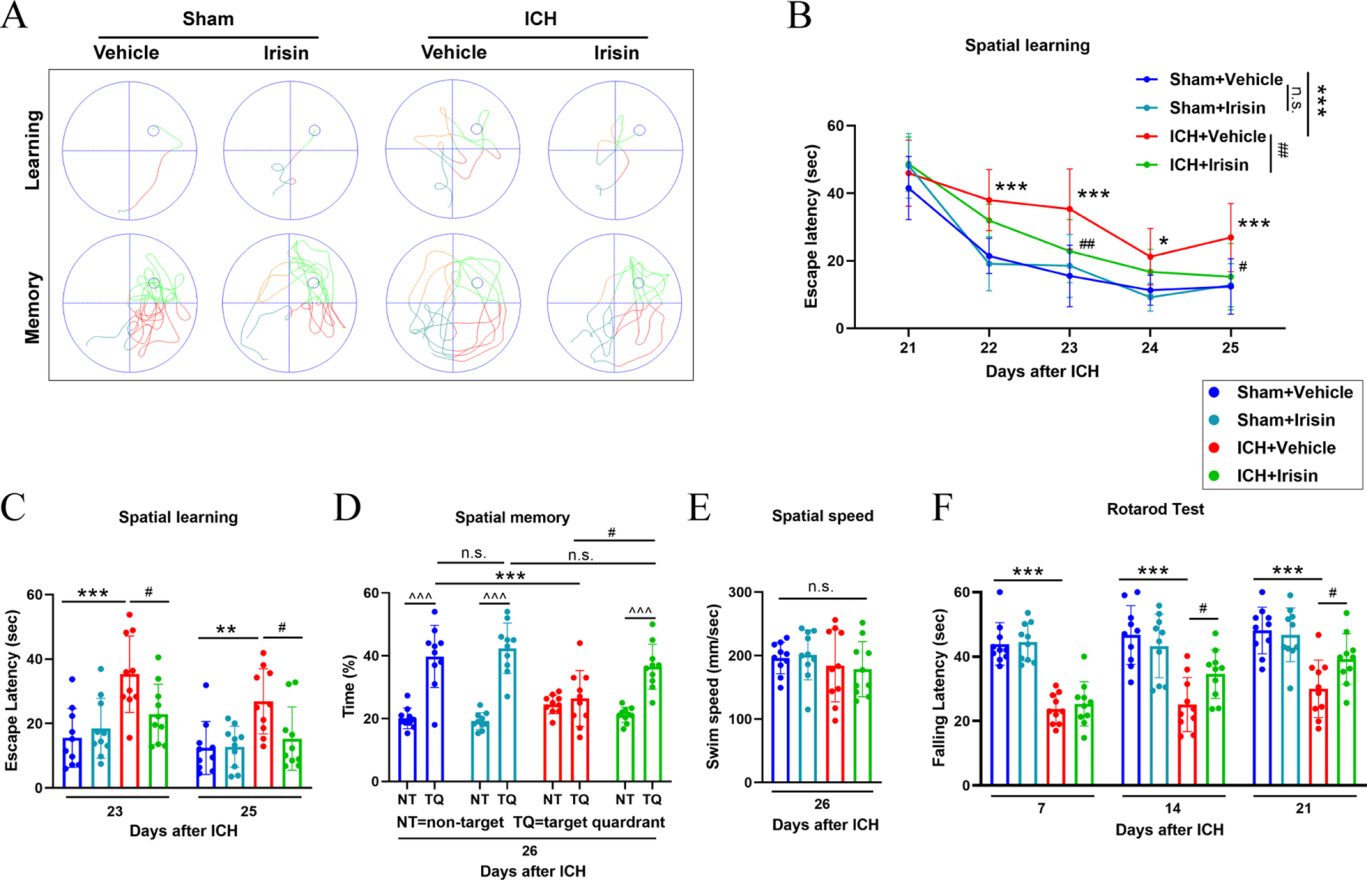
Effects of irisin on long-term neurobehavioral outcomes after ICH. **A** Representative swim paths of Morris water maze during the learning and memory phases of the test. **B**, **C** Spatial learning was assessed by the escape latency of Morris water maze on days 21–25 after ICH. **D** Spatial memory was assessed by the time spent in the target quadrant on day 26 after ICH. **E** Average swim speed for mice in the Morris water maze. **F** Rotarod test on days 7, 14, and 21 post-ICH. *p < 0.05, **p < 0.01, ***p < 0.001 vs. sham + vehicle group; ^#^*p* < 0.05, ^##^*p* < 0.01 vs. ICH + vehicle group; ^*p* < 0.05, ^^*p* < 0.01, ^^^*p* < 0.001 vs. NT. Error bars are represented as mean ± SD. *n* = 10 per group

To study the long-term effects of irisin treatment on ICH, we performed the Morris water maze (MWM) test and Rotarod test to evaluate post-ICH spatial cognitive functions and motor functions, respectively. In the Rotarod test, the mice in ICH + vehicle group had significantly shorter falling latency when compared with sham groups at days 7, 14, and 21 post-ICH (*p* < 0.001, Fig. 3F). However, irisin post-treatment significantly improved the neurological deficits when compared with the ICH + vehicle group on days 14 (*p* < 0.05, Fig. 3F) and 21 (*p* < 0.05, Fig. 3F) after ICH. Spatial learning and memory were evaluated at 21–26 days after ICH by the MWM test. The results of the MWM test did not reveal any significant differences between irisin and vehicle-treated administration in sham mice (Fig. 3B). Thus, it can be concluded that irisin post-treatment does not have any negative side effects on sham mice. In addition, the MWM test revealed that the escape latency was progressively decreased in all tested groups during the spatial learning period at 21–25 days after ICH (Fig. 3B). However, the ICH + vehicle group showed a significantly longer escape latency compared to the vehicle-treated sham group (*p* < 0.001, Fig. 3B). ICH + vehicle group showed a significantly higher escape latency compared to the vehicle-treated sham group especially on day 23 (*p* < 0.001, Fig. 3C) and day 25 (*p* < 0.01, Fig. 3C) post-ICH. In contrast, ICH + irisin mice demonstrated a significant diminished escape latency on days 23 (*p* < 0.05, Fig. 3C) and 25 (*p* < 0.05, Fig. 3C) compared to vehicle-treated ICH mice. To further provide evidence for spatial memory deficits, a probe quadrant trial was performed on day 26, where the mice were tested without a platform to escape. Here, we compared the mean time in percentage of the three nontarget quadrants (NT) to the percent time spent in the target quadrant (TQ, where the platform was located during the spatial learning period). The results showed a significant preference for the TQ in sham and ICH + irisin mice (*p* < 0.001, Fig. 3D), whereas no TQ preference was detected in vehicle-treated ICH mice (Fig. 3D). Besides, irisin-treatment markedly increased the time spent in the probe quadrant compared with the ICH + vehicle group (*p* < 0.05, Fig. 3D). This was visualized with the representative swim paths which were chaotic post-ICH but improved by irisin post-treatment (*p* < 0.01, Fig. 3A, B). All mice had similar swim speeds (Fig. 3E), reflecting comparable locomotor functions among all test groups. Taken together, these data confirmed an impaired spatial learning and memory function in post-ICH mice, which can be markedly ameliorated following irisin post-treatment.

### Irisin treatment alleviated neuronal apoptosis after 24 h after ICH

**Fig. 4:**
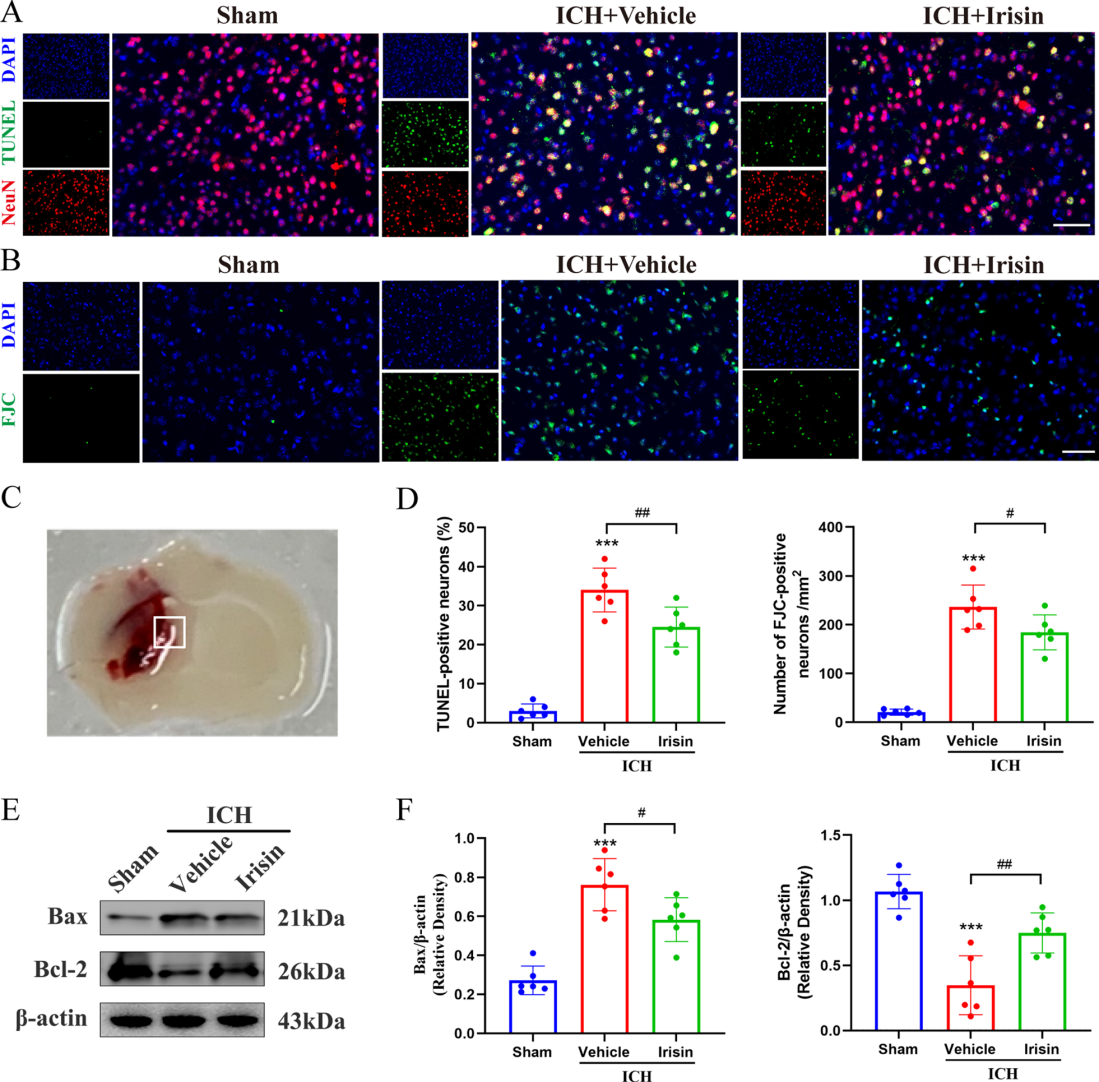
Effects of irisin post-treatment on neuronal apoptosis and neuronal apoptotic molecular markers after ICH. **A**, **B** Representative images of the co-localization of TUNEL (green) with neurons (NeuN, red) and FJC (green) staining in the perihematomal area at 24 h after ICH. **C** Brain sample with schematic illustration showing the area (indicated by the white square) used for TUNEL and FJC-positive cell counting in the perihematomal region. **D** Quantitative analyses of TUNEL and FJC-positive cells in the perihematomal area at 24 h after ICH (*n* = 6/group). **E**, **F** Representative western blot bands and quantitative analyses of Bax and Bcl-2 protein levels at 24 h after ICH. *p < 0.05, **p < 0.01, ***p < 0.001 vs. sham group; ^#^*p* < 0.05, ^##^*p* < 0.01 vs. ICH + vehicle group, mean ± SD, one-way ANOVA, Tukey test, *n* = 6/group, scale bar = 50 μm

To assess the effects of irisin post-treatment in neuronal death after ICH, apoptotic and degenerating neurons in the perihematomal area at 24 h after ICH was assessed by TUNEL and FJC staining. TUNEL-positive and FJC-positive neurons were significantly increased in the perihematomal region of the vehicle-treated ICH group compared to the sham group at 24 h post-ICH (*p* < 0.001, Fig. 4A–D). However, irisin administration decreased the number of TUNEL-positive (*p* < 0.01, Fig. 4A, D) and FJC-positive (*p* < 0.05, Fig. 4B, D) neurons. Meanwhile, the expression of neuronal apoptotic molecular markers Bax and Bcl-2 at 24 h after ICH was measured by western blot analysis. Consistently, the results revealed that irisin post-treatment in the ipsilateral hemisphere led to a significant increase in Bcl-2 (pro-survival marker) expression (*p* < 0.05, Fig. 4E, F), while Bax (pro-apoptotic marker) levels (*p* < 0.01, Fig. 4E, F) were markedly reduced compared to the vehicle-treated ICH group at 24 h post-ICH.

### Irisin treatment inhibited microglia/macrophage activation, neutrophil infiltration, and the expression of IL-1β at 24 h after ICH

**Fig. 5:**
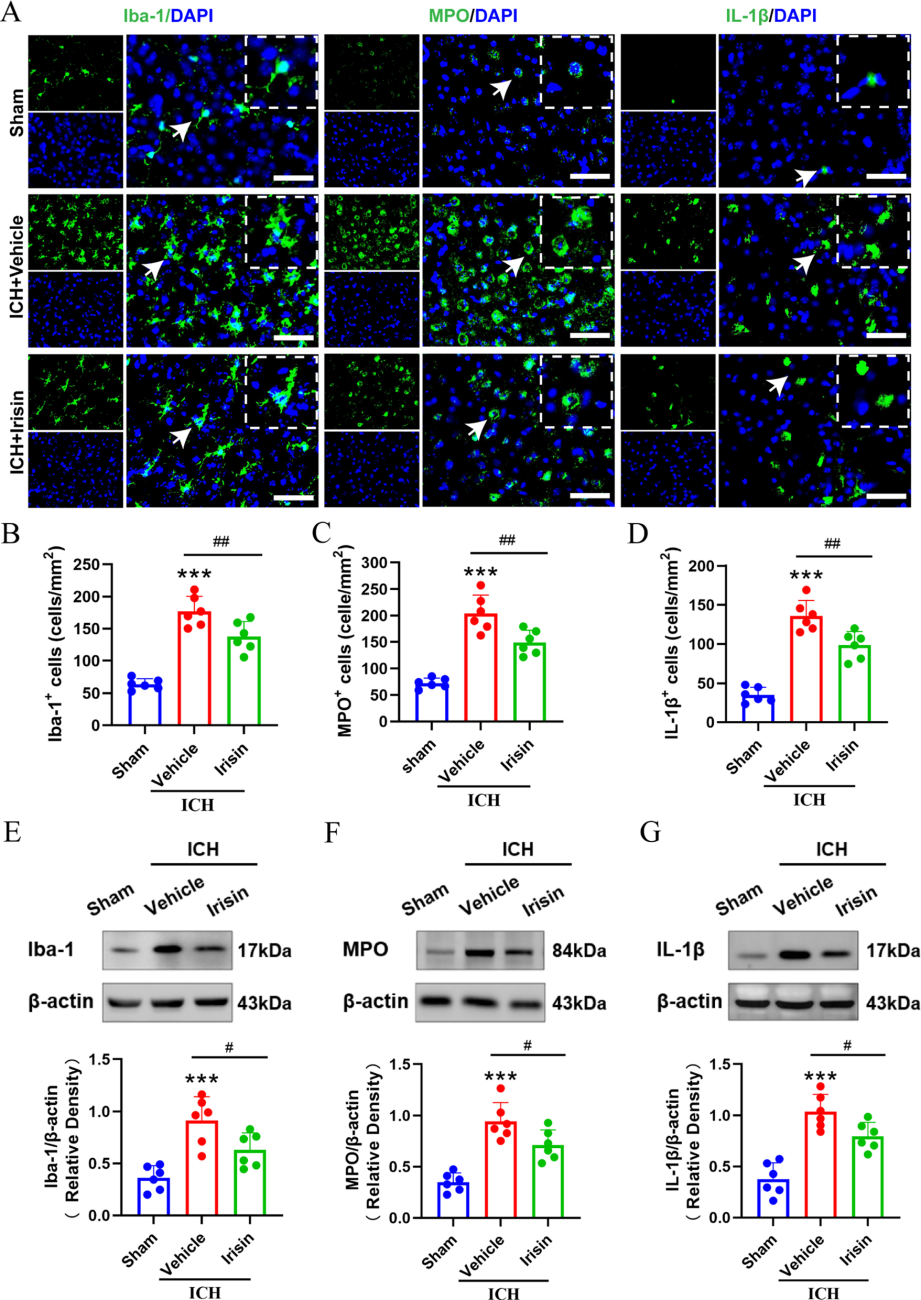
Effects of irisin on microglia/macrophage activation and neutrophil infiltration after ICH. **A** Representative images of immunofluorescence staining of Iba-1 (green), MPO (green), and IL-1β (green) in the perihematomal area at 24 h after ICH. **B**–**D** Quantitative analyses of Iba-1, MPO, and IL-1β-positive cells in the perihematomal area at 24 h after ICH, *n* = 6 per group. **E**–**G** Representative Western blot bands and quantitative analyses of Iba-1, MPO, and IL-1β protein levels in the ipsilateral hemisphere at 24 h after ICH. *p < 0.05, **p < 0.01, ***p < 0.001 vs. sham group; ^#^*p* < 0.05, ^##^*p* < 0.01 vs. ICH + vehicle group. Error bars are represented as mean ± SD. One-way ANOVA, Tukey’s test, *n* = 6 per group, scale bar = 50 μm

At 24 h post-ICH, the levels of Iba-1 and MPO in the perihematomal area were performed by immunofluorescence staining to detect microglia/macrophage activation and neutrophil infiltration. Immunofluorescence staining showed that the numbers of Iba-1, IL-1β, and MPO-positive cells were significantly increased in ICH + vehicle group when compared with the sham group (*p* < 0.001, Fig. 5A–D). However, irisin post-treatment significantly reduced the number of Iba-1 (*p* < 0.05, Fig. 5A, B), MPO (*p* < 0.01, Fig. 5A, C), or IL-1β-positive cells (*p* < 0.01, Fig. 5A, D) in the perihematomal area than that in ICH + vehicle group. In addition, western blot results showed that the expression of Iba-1, MPO, and IL-1β in the ipsilateral hemisphere were significantly decreased with irisin post-treatment when compared with ICH + vehicle group at 24 h after ICH (*p* < 0.05, Fig. 5E–G).

### Irisin treatment increases the phenotypic switch of microglia/macrophage from pro-inflammatory to an anti-inflammatory phenotype

**Fig. 6:**
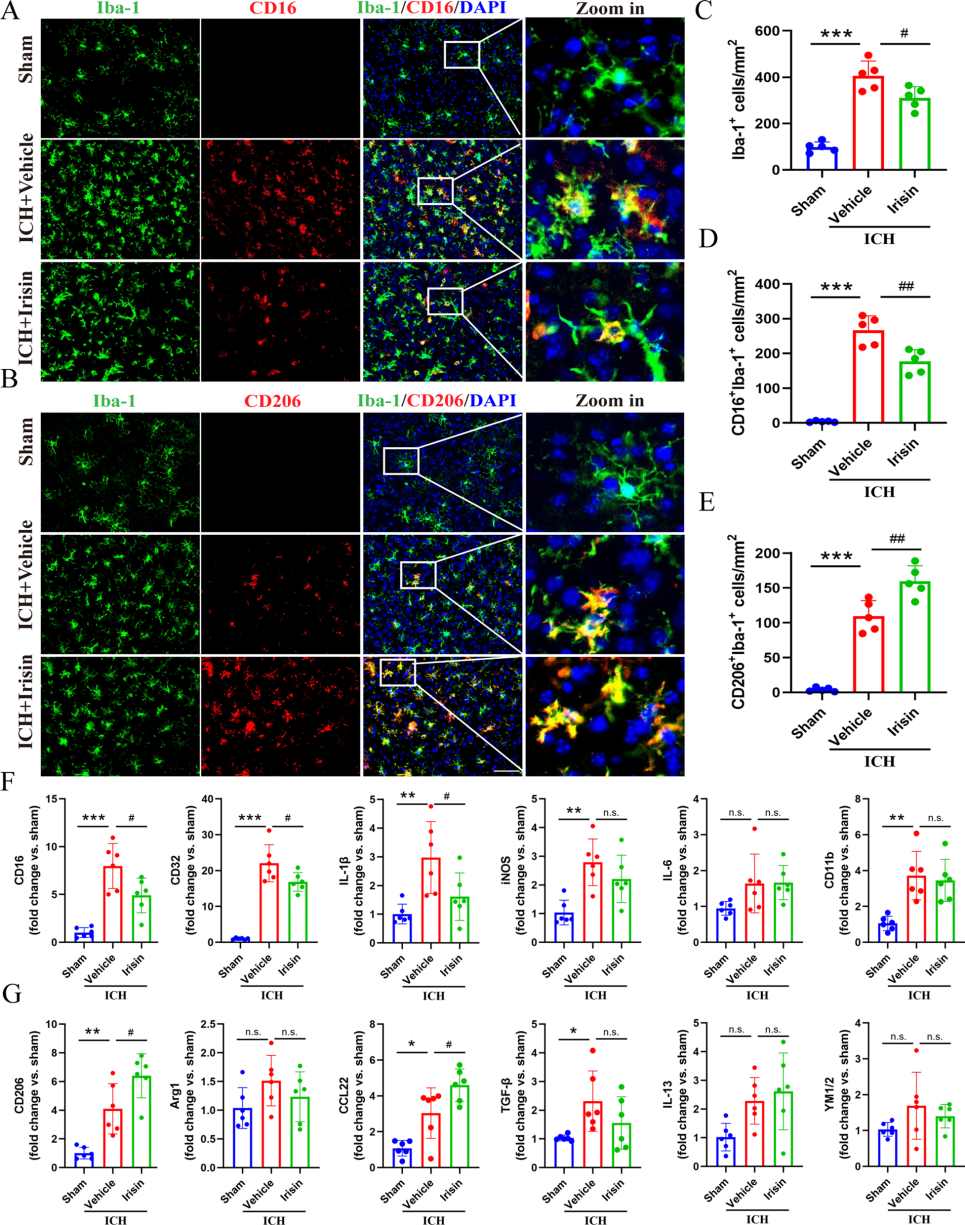
Irisin promotes the phenotype of microglia/macrophage from pro-inflammatory into anti-inflammatory at 72 h post-ICH. **A** Representative double immunofluorescence staining for Iba1 (green) and CD16 (red) in the perihematomal area. Scale bar = 50 μm. **B** Representative images of Iba1 (green) and CD206 (red) immunostaining in the perihematomal area. Scale bar = 50 μm. **C**–**E** Quantitative analyses of Iba1^+^ microglia/macrophage, Iba1^+^/CD16^+^ M1 microglia/macrophage and Iba1^+^/CD206^+^ M2 microglia/macrophage in the perihematomal area at 72 h after ICH. **F** M1-associated mRNA levels were evaluated including CD16, CD32, IL-1β, iNOS, IL-6, and CD11b. **G** M2-associated mRNA levels were evaluated including CD206, Arg1, CCL22, TGF-β, IL-13, and YM1/2. Data were represented as mean ± SD. *p < 0.05, **p < 0.01, ***p < 0.001 vs. sham group; ^#^*p* < 0.05, ^##^*p* < 0.01 vs. ICH + vehicle group. One-way ANOVA, Tukey test, *n* = 6/group

In response to acute brain injury, microglia become activated and develop classic pro-inflammatory (M1) or anti-inflammatory (M2) phenotypes. The different phenotypes of microglia influence the outcome of ICH. We performed immunofluorescence staining to confirm whether irisin affects microglia/macrophage phenotypic switch. The numbers of the Iba-1^+^ microglia were significantly reduced in the perihematomal area of the ICH + irisin group compared with the ICH + vehicle group (*p* < 0.05, Fig. 6A–C), which suggested that irisin alleviated microglial activation at 72 h post-ICH. Comparing with the ICH + vehicle group, we found that irisin post-treatment significantly decreased the number of Iba1^+^CD16^+^ M1 microglia/macrophages (*p* < 0.01, Fig. 6D) and significantly increased the numbers of Iba1^+^CD206^+^ M2 microglia/macrophages (*p* < 0.01, Fig. 6E) in the perihematomal area at 72 h post-ICH. To further investigate the phenotype characteristics of microglia/macrophages, qPCR was conducted to evaluate the mRNA expression levels of M1 phenotype markers (CD16, CD32, IL-1β, iNOS, IL-6, and CD11b) and M2 phenotype markers (CD206, Arg1, CCL22, TGF-β, IL-13, and YM1/2). Compared with the vehicle group, mice treated with irisin showed lower expression of M1 markers (CD16, CD32, and IL-1β) and higher expression of M2 markers (CD206 and CCL22) in the perihematomal region (*p* < 0.05, Fig. 6F, G). Taken together, these findings showed that irisin post-treatment increased the phenotypic switch of microglia/macrophages from pro-inflammatory to anti-inflammatory phenotype after ICH.

### Integrin αVβ5 inhibitor abolished the neuroprotection of irisin on neurological functions at 24 h after ICH

**Fig. 7:**
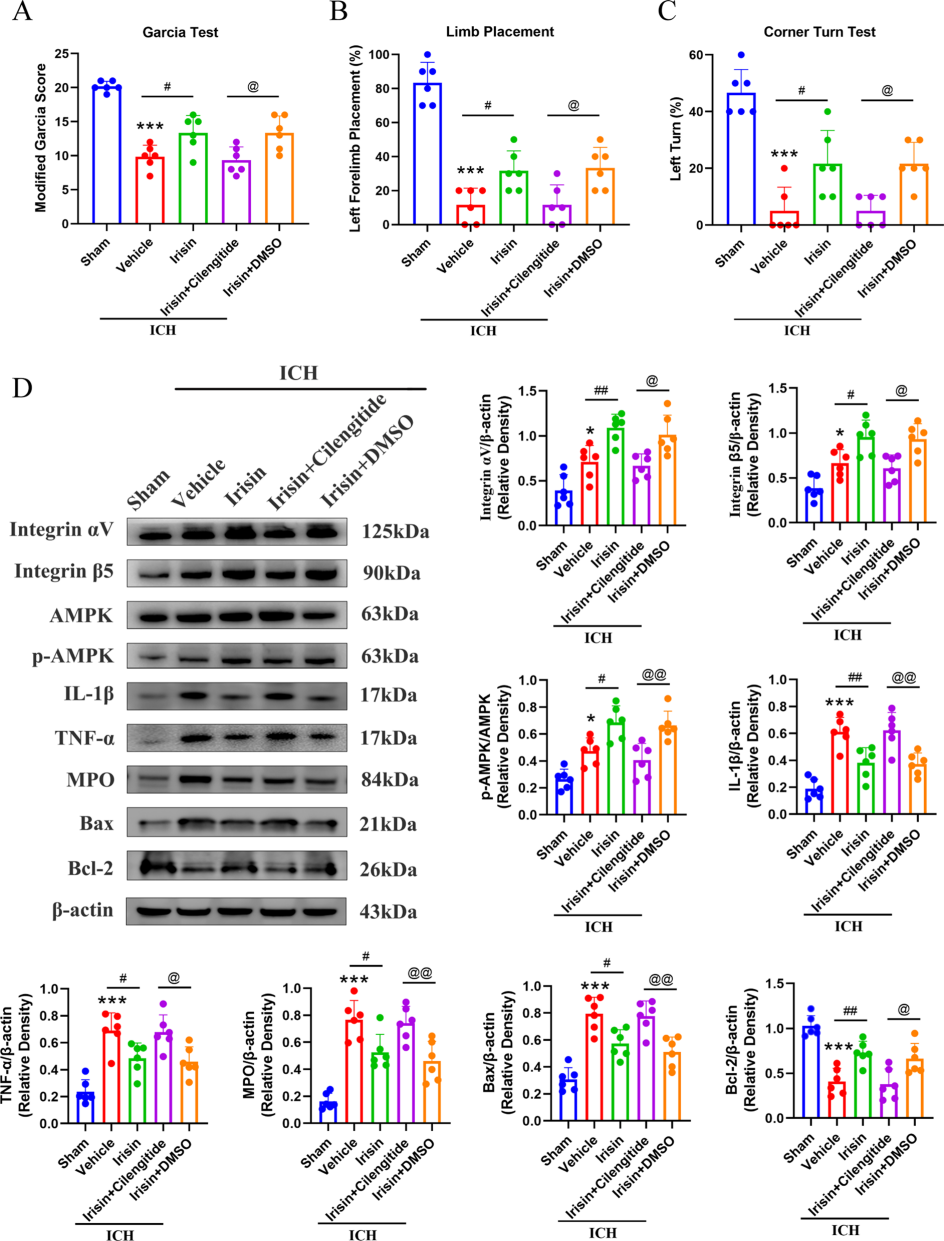
Integrin αVβ5 inhibition reverses the effects of irisin on neurological functions and inflammatory/apoptotic proteins expression at 24 h after ICH. **A**–**C** Modified Garcia test, forelimb placement test, and corner turn test. **D** Representative western blot images and quantitative analyses of integrin αV, integrin β5, p-AMPK/AMPK, IL-1β, TNF-α, MPO, Bax and Bcl-2 at 24 h post-ICH. Values are expressed as mean ± SD. *p < 0.05, **p < 0.01, ***p < 0.001 vs. sham group; ^#^*p* < 0.05, ^##^*p* < 0.01 vs. ICH + vehicle group; ^@^*p* < 0.05, ^@@^*p* < 0.01 vs. irisin + DMSO group. Data was represented as mean ± SD. One-way ANOVA, Tukey test, *n* = 6/group

To understand the mechanism of the neuroprotection of irisin, we first analyze whether integrin αVβ5 receptor was involved in the neuroprotective effects of irisin, and we inhibited integrin αVβ5 receptor with cilengitide. Pre-administration of integrin αVβ5 inhibitor cilengitide significantly aggravated the neurobehavioral benefits of irisin assessed by the modified Garcia (*p* < 0.05, Fig. 7A), forelimb placement (*p* < 0.05, Fig. 7B), and corn turn tests (*p* < 0.05, Fig. 7C) in ICH + irisin + cilengitide group when compared with ICH + irisin + DMSO group at 24 h after ICH. The expression of integrin αV, integrin β5, p-AMPK, IL-1β, TNF-α, MPO, and Bax was significantly increased, while the expression of Bcl-2 was remarkably decreased when compared with the sham group at 24 h after ICH (*p* < 0.05, Fig. 7D). Irisin post-treatment significantly increased the expression of integrin αV, integrin β5, p-AMPK, Bcl-2 and decreased the expression of IL-1β, TNF-α, MPO, and Bax in ICH + irisin group when compared with ICH + vehicle group at 24 h after ICH (*p* < 0.05, Fig. 7D). However, inhibition of integrin αVβ5 with cilengitide remarkably decreased the expression of integrin αV, integrin β5, p-AMPK, Bcl-2 and increased the expression of IL-1β, TNF-α, MPO, and Bax in ICH + irisin + cilengitide group when compared with ICH + irisin + DMSO group at 24 h after ICH (*p* < 0.05, Fig. 7D).

### AMPK inhibitor abolished the neuroprotective effects of irisin on neurological functions at 24 h after ICH

**Fig. 8:**
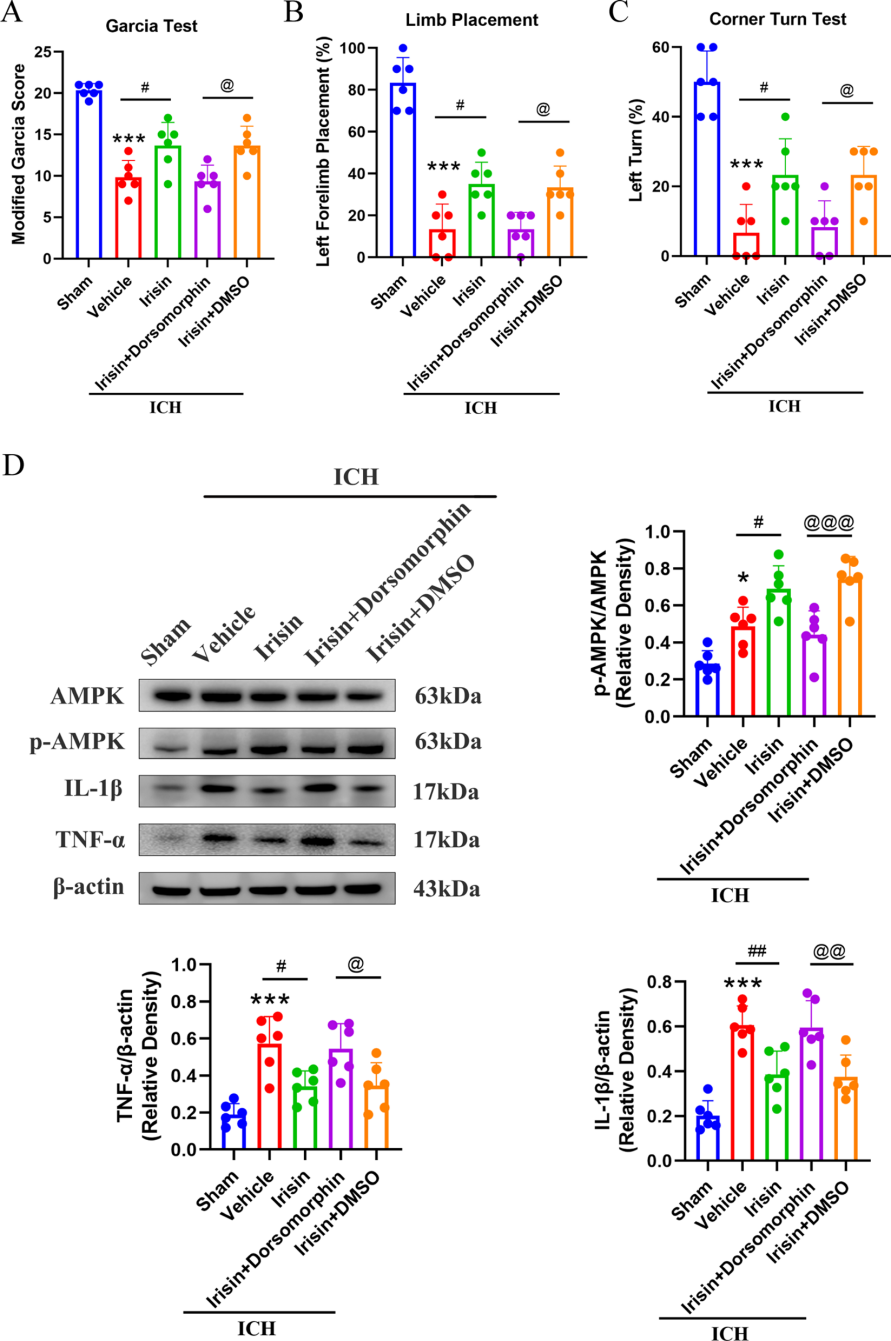
AMPK inhibition reverses the effects of irisin on neurological functions and inflammatory proteins expression at 24 h after ICH. **A**–**C** Modified Garcia test, forelimb placement test, and corner turn test. **D** Representative western blot images and quantitative analyses of p-AMPK/AMPK, IL-1β, TNF-α at 24 h post-ICH. Values are expressed as mean ± SD. *p < 0.05, **p < 0.01, ***p < 0.001 vs. sham group; ^#^*p* < 0.05, ^##^*p* < 0.01 vs. ICH + vehicle group; ^@^*p* < 0.05, ^@@^*p* < 0.01 vs. irisin + DMSO group. Data were represented as mean ± SD. One-way ANOVA, Tukey test, *n* = 6/group

To assess whether the AMPK signaling pathway was involved in the neuroprotective and anti-inflammatory effects of irisin, AMPK inhibitor dorsomorphin was applied. Intracerebroventricular injection of dorsomorphin, a selective AMPK inhibitor, significantly abolished the neurobehavioral improvements of irisin post-treatment in the modified Garcia (*p* < 0.05, Fig. 8A), forelimb placement (*p* < 0.05, Fig. 8B), and corn turn tests (*p* < 0.05, Fig. 8C) in ICH + irisin + dorsomorphin group when compared with ICH + irisin + DMSO group at 24 h after ICH. Western blot analysis revealed that the expression of p-AMPK, IL-1β, and TNF-α was significantly increased when compared with the sham group at 24 h after ICH (*p* < 0.05, Fig. 8D). Irisin post-treatment significantly increased the expression of p-AMPK while remarkably decreasing the expression of IL-1β and TNF-α in the ICH + irisin group when compared with ICH + vehicle group at 24 h after ICH (*p* < 0.05, Fig. 8D). However, pretreatment with dorsomorphin, a selective AMPK inhibitor, remarkably decreased the expression of p-AMPK and increased the expression of IL-1β, TNF-α in ICH + irisin + dorsomorphin group when compared with ICH + irisin + DMSO group at 24 h after ICH (*p* < 0.05, Fig. 8D).

## Discussion

In the current study, we investigated the neuroprotective effects of irisin-dependent integrin αVβ5 activation and explored the underlying mechanism in a mouse model of ICH. For the first time, we demonstrated that the expression of irisin decreased at 3 h and increased to the peak at 24 h after ICH. As a functional receptor of irisin, integrin αVβ5 is mainly expressed in the microglia/macrophages, and it showed a similar time course expression with irisin after ICH. Moreover, the activation of integrin αVβ5 with irisin significantly improved the short-term and long-term neurobehavioral deficits, accompanied by reduced brain edema, attenuated microglia/macrophage activation, increased microglia/macrophage phenotypic switch from pro-inflammatory to anti-inflammatory phenotypes, and inhibited peripheral neutrophil infiltration in perihematomal areas after ICH. Mechanistically, irisin treatment upregulated the protein levels of p-AMPK, Bcl-2, but downregulated the protein levels of MPO, IL-1β, TNF-α, and Bax within the ipsilateral hemisphere at 24 h after ICH. In addition, integrin αVβ5 inhibitor cilengitide or selective AMPK inhibitor dorsomorphin abolished the beneficial effects of irisin on neurological deficits and neuroinflammation. Taken together, our findings suggest that irisin could enhance the activation of integrin αVβ5 in microglia/macrophages at 24 h after ICH and played an important role in ameliorating neuroinflammation and neuronal apoptosis, partially by up-regulating the integrin αVβ5/AMPK signaling pathway. The administration of irisin may serve as an effective therapeutic strategy against ICH-induced SBI after ICH.

Irisin is a secreted portion released from FNDC5, a transmembrane precursor protein mainly expressed in muscle. As a newly identified myokine first discovered in 2012, irisin is released into the circulation during physical exercise and is initially recognized as stimulating adipocyte browning and thermogenesis in humans and mice [15]. The following studies have reported the existence of irisin in the brain regions, such as the hippocampus, cerebrum, and even in cerebrospinal fluid [16–18]. It is reported that serum level of irisin was decreased upon cerebral ischemic stress [21], and the protein level of irisin in the ipsilateral hemisphere was increased at 12 h while decreased at 24 h after subarachnoid hemorrhage injury [35]. However, in this study, we found that the level of irisin in the serum decreased significantly after ICH within 24 h, and the endogenous expression of irisin in the ipsilateral/right hemisphere of the brain was decreased at 3 h and increased to the peak at 24 h after ICH. There may be some reasons accounting for that. First, the self-regulating system of the body would be affected by ICH stress to redistribute the endogenous hormones and accelerate irisin crossing the BBB. Second, the physical movement of the post-ICH mice would be reduced and thus the serum irisin level would be decreased. Even though the endogenous irisin increased after ICH, it may not be sufficient to significantly exert neuroprotection post-ICH. In this study, we administered recombinant irisin by the intranasal route. Intranasal administration is an easy and non-invasive method that permits the delivery of drugs bypassing BBB to the central nervous system (CNS). In addition, this is the first study to use irisin by the intranasal route. There is growing evidence that supports neuroprotective drugs could be successfully delivered to the brain via the nasal route [45].

Recently, the anti-inflammatory, anti-apoptotic and anti-oxidative properties of irisin have received a great deal of attention in various diseases [22]. Previous studies explored the critical role that irisin played in ischemic stroke in mice models, indicating that irisin can upregulate the levels of brain-derived neurotrophic factor (BDNF) and protects nerve cells from injury during ischemic stroke [46, 47]. Clinical investigations revealed that decreased serum concentration of irisin is associated with poor functional outcomes and post-stroke depression in ischemic stroke patients [48, 49]. For the first time, our research reported that irisin improved short- and long-term neurobehavioral deficits, decreased brain edema, attenuated microglia/macrophage activation, and neutrophil infiltration, and downregulated the expression of proinflammatory cytokines TNF-α and IL-1β in perihematomal areas after ICH.

However, the mechanisms underlying the benefits of irisin were unclear, on a large scale, because the irisin receptor(s) had not been identified. A previous study reported that irisin could bind to the integrin αVβ5 receptor on osteocytes and fat cells, and a specific αVβ5 inhibitor could block the signaling and function by irisin in osteocytes and fat cells [23]. This is the very first finding in which the irisin receptor was identified. Interestingly, the integrin αVβ5 receptor comprises the major fraction of total αV integrins expressed by the microglia/macrophages [25]. Our research further showed that irisin was co-localized with integrin αVβ5 on the microglia/macrophages, which indicated that irisin could bind to integrin αVβ5 receptor on the microglia/macrophages. In addition, the endogenous expression of integrin αVβ5 showed a similar time course expression to that of the irisin after ICH.

Current scientific research indicated that the primary brain injury after ICH could be the mass effect of the hematoma. Besides, a cascade of multifactorial events caused by the components of the red cells (hemoglobin and iron) could accelerate the SBI, which is a vital process leading to poor prognosis post-ICH. Neuroinflammation is a crucial pathophysiological component leading to SBI after ICH [6]. Uncontrolled neuroinflammation is a major cause for the increased apoptosis and dysfunction of various brain cells (e.g., neurons and endothelial cells), thus exacerbating the neurological outcome of ICH [50]. Microglia, the classical resident immunocytes of the CNS, react to various brain injury and plays an important role in neuroinflammation within the CNS. Besides, peripheral monocyte-derived macrophages (MDMs) are recruited in the early hours post-ICH, and they mature into macrophages in the CNS parenchyma. Similar cellular surface markers including CD11b and Iba-1 are expressed on the brain-derived microglia and peripheral macrophages and it is hard to distinguish between them [51]. Our data showed that the number of Iba-1^+^ microglial cells in the perihematomal area at 24 h after ICH was significantly increased compared to the sham group. Besides, MPO^+^ staining suggested that peripheral neutrophil infiltration was increased in the brain parenchyma, with the inflammatory cytokine IL-1β showing the same trend. Western blot analysis performed for further quantitative analysis also manifested that the protein expression levels of Iba-1, MPO, and IL-1β in the perihematomal area post-ICH were significantly increased.

It has been reported that microglia/macrophage respond to acute brain injury through becoming activated and can polarize into two phenotypes: classically activated (M1-like) or alternatively activated (M2-like) and this phenotype changes dynamically [52]. Classically activated M1 microglia/macrophage phenotype mainly produces cytokines, chemokines, and other pro-inflammatory factors, which accelerate tissue damage. On the contrary, alternatively accelerated M2 microglia/macrophage mainly produces anti-inflammatory factors and clears up cell debris. Though recent literature questions the effectiveness of this nomenclature, since microglial/macrophage response to the brain injury is too dynamic and complex to be defined by the concept of polarization, the dichotomy between M1 and M2 phenotypes classification remains useful for clarifying the character of microglia/macrophage in various brain disorders [53]. Here, we confirmed that irisin administration significantly reduced pro-inflammatory microglia/macrophage and increased anti-inflammatory microglia/macrophage at 72 h after ICH. The expressions of the markers corroborated the effect of recombinant irisin on microglia/macrophage, providing exact evidence for the anti-inflammatory properties of irisin in ICH. Mounting evidence has indicated that the promotion of a switch from M1 to M2 phenotype could ameliorate SBI and improve neurological functions after ICH [52]. Moreover, timing the most efficient switch of M1 to M2 phenotype should be cautiously considered, hence it is vital to evaluate the time course of M1 and M2-polarization after ICH. It’s reported that the M1 to M2 phenotypic switch mainly occurs within the first 7-day post-ICH [54]. Moreover, Lan et al. [55] indicated that M1 microglia/macrophage polarization might be the main cause of microglial/macrophage activation in the acute phase after ICH, whereas M2-like microglial/macrophage responses and mediators might have an important role in the subacute and chronic phase and might contribute to phagocytosis of cell debris and hematoma clearance. Therapeutically, efficient tissue repair requires the cooperation of both M1 and M2 phenotype microglial/macrophages, and the best time switches in microglial/macrophage phenotype should be considered.

Furthermore, we explored the possible mechanism underlying the irisin-dependent integrin αVβ5 activation-mediated neuroinflammation after ICH. Previous studies revealed that phosphorylation of AMPK could mitigate cell apoptosis and alleviate neuroinflammation in various pathophysiological processes [26–29]. It is also reported that AMPK activation is an important mechanism underlying the inhibition of macrophage/microglia-mediated inflammation [56]. Moreover, irisin could restore gut barrier function after ischemia–reperfusion via activation of the integrin αVβ5-AMPK pathway [24]. Our results demonstrated that irisin post-treatment markedly increased the expression of p-AMPK as well as downregulated the expressions of pro-inflammatory cytokines including IL-1β and TNF-α at 24 h after ICH. Cilengitide trifluoroacetate, an inhibitor of integrin αVβ5 [24], was shown to significantly decrease the expression of p-AMPK and reversed the beneficial effects of irisin on neurobehavior deficits and neuroinflammation after ICH. In addition, our data showed that dorsomorphin, a selective phosphorylated AMPK inhibitor [3] significantly reversed the anti-inflammatory, anti-apoptotic effects and neurobehavior protection of irisin post-treatment. Collectively, these data suggested that irisin post-treatment alleviated neuroinflammation possibly through the integrin αVβ5/AMPK signaling pathway after ICH.

Several limitations should be pointed out in this study. First, the present study mainly focused on irisin-mediated neuroinflammation post-ICH. Future studies are required to explore the other mechanisms underlying the neuroprotective effects of irisin against SBI after ICH, such as BBB disruption. Second, since the pathophysiology of neuroinflammation after ICH is a complex network, the contribution of PI3k/Akt signaling in irisin-mediated inflammation cannot be excluded. Third, although the intranasal administration route assured the neuroprotective role of irisin played in CNS, crosstalk of plasma concentration and brain parenchyma level of irisin should be further illustrated. Besides, the muscle–brain crosstalk which influences the relevance of brain and plasma levels of irisin remains to be further investigated. Finally, we did not evaluate the sex- and age-specific differences in the effects of irisin after ICH. Emerging evidence indicated that sex and age are two vital risk factors determining the prognosis after ICH [57, 58]. Estrogen has been reported to reduce neurological impairment after ICH in rats [59]. Previous studies showed that improved neurobehavioral outcomes and reduced mortality occur in females compared to male mice post-ICH [60]. Besides, ICH tends to occur in the elderly population with cerebrovascular disease and hypertension [60]. In the present study, we only used 8-week-old male adult mice for our experiments. We did not evaluate the effects of irisin in different age groups or ICH with systemic co-morbidities, and female animals. Therefore, further studies are needed to certify the neuroprotective effects of irisin in experimental ICH in different age groups and females.

## Conclusions

In summary, we first showed that irisin administration ameliorated neurological deficits, reduced brain edema, ameliorated neuroinflammation, and neuronal apoptosis, at least in part, through the integrin αVβ5/AMPK signaling pathway after ICH. Thus, irisin post-treatment may provide a promising therapeutic approach for the early management of ICH.

## Supplementary Information

**Additional file 1. Supplementary Fig. S1**. Experimental design and animal groups. ICH, intracerebral hemorrhage; WB, western blot; TUNEL, transferase dUTP nick end labeling; ELISA, enzyme-linked immunosorbent assay; qPCR, quantitative real-time polymerase chain reaction.** Supplementary Table S1**. Summary of experimental groups and mortality rate in the study.** Supplementary Fig. S2**. Representative double immunofluorescence staining for Iba-1 (magenta) and irisin (green) in sham group and the perihematomal area of ICH (24 h) group. Scale bar = 100 um.** Supplementary Fig. S3**. Representative western blot bands and quantitative analyses of time course of irisin expression in the ipsilateral hemisphere after ICH in vehicle and irisin-treated ICH groups. #*p* < 0.05, ##*p* < 0.01, ###*p* < 0.001 vs. ICH+Vehicle group, mean±SD, n = 6/group.

## Data Availability

The data, analytic methods, and study materials will be made available to other researchers to reproduce the results or replicate the procedures. The data that support the findings of this study are available from the corresponding author upon reasonable request. Authors will be responsible for maintaining availability.

## Abbreviations

ICH: Intracerebral hemorrhage
AMPK: Adenosine monophosphate-activated protein kinase
MPO: Myeloperoxidase
IL-1β: Interleukin-1β
TNF-α: Tumor necrosis factor α
i.n.: Intranasal
i.p.: Intraperitoneal
DMSO: Dimethylsulfoxide
GFAP: Glial fibrillary acidic protein
Iba-1: Ionized calcium-binding adaptor molecule-1
NeuN: Neuronal nuclear
p-AMPK: Phosphorylated-AMPK
PBS: Phosphate-buffered saline
i.c.v.: Intracerebroventricular
BWC: Brain water content
WW: Wet weight
DW: Dry weight
FJC: Fluoro-Jade C
TUNEL: Terminal deoxynucleotidyl transferase dUTP nick end labeling
CNS: Central nervous system
BBB: Blood–brain barrier
