# Supplementary material for: Irisin ameliorates neuroinflammation and neuronal apoptosis through integrin αVβ5/AMPK signaling pathway after intracerebral hemorrhage in mice
Source: J Neuroinflammation. 2022 Apr 7;19:82. doi: 10.1186/s12974-022-02438-6 (PMC8988353; doi:10.1186/s12974-022-02438-6)
Supplement: Supplementary file 1 — Additional file 1. Supplementary Fig. S1. Experimental design and animal groups. ICH, intracerebral hemorrhage; WB, western blot; TUNEL, transferase dUTP nick end labeling; ELISA, enzyme-linked immunosorbent assay; qPCR, quantitative real-time polymerase chain reaction. Supplementary Table S1. Summary of experimental groups and mortality rate in the study. Supplementary Fig. S2. Representative double immunofluorescence staining for Iba-1 (magenta) and irisin (green) in sham group and the perihematomal area of ICH (24 h) group. Scale bar = 100 um. Supplementary Fig. S3. Representative western blot bands and quantitative analyses of time course of irisin expression in the ipsilateral hemisphere after ICH in vehicle and irisin-treated ICH groups. #p < 0.05, ##p < 0.01, ###p < 0.001 vs. ICH+Vehicle group, mean±SD, n = 6/group. [file 12974_2022_2438_MOESM1_ESM.docx]

**Supplementary materials**

**Part 1: Experimental design**

In the present study, all mice were randomly assigned to the following experiments.


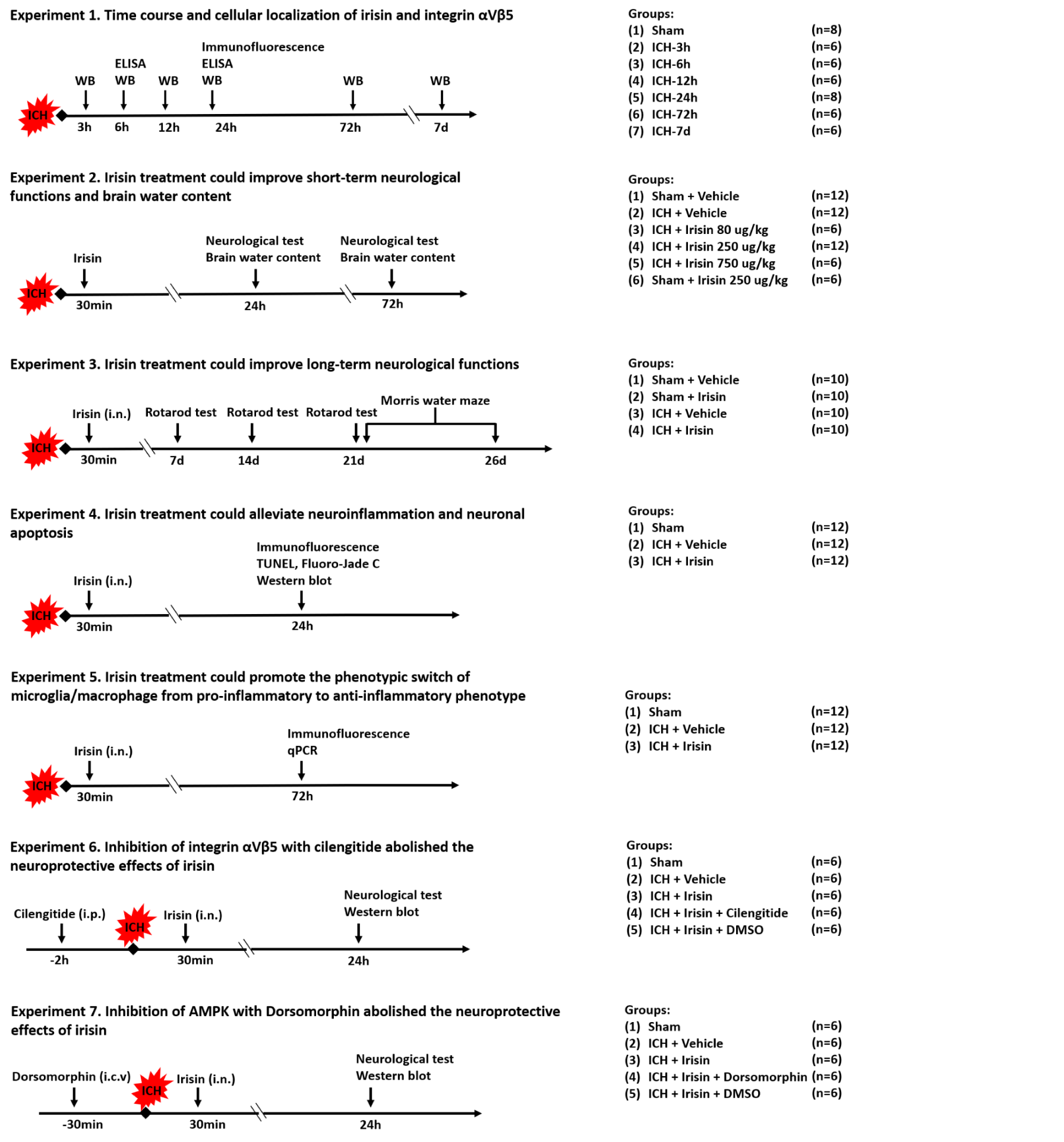


Figure S1. Experimental design and animal groups. ICH, intracerebral hemorrhage; WB, western blot; TUNEL, transferase dUTP nick end labeling; ELISA, enzyme-linked immunosorbent assay; qPCR, quantitative real-time polymerase chain reaction.

**Experiment 1**

To evaluate the time course of endogenous irisin and integrin αVβ5 expression in the ipsilateral/right hemisphere post-ICH, 42 mice were randomly assigned to seven groups (n = 6/group): sham, 3, 6, 12, 24, 72 h and 7 days after ICH for western blot analysis. Enzyme-linked Immunosorbent Assay (ELISA) was performed to evaluate the time course of plasma levels of irisin. Additional four mice (n = 2/group) were used for immunofluorescence staining at 24 h post-ICH. The cellular localization of integrin αVβ5 was assessed using double-labeling immunofluorescence staining to co-localize integrin αVβ5 with ionized calcium binding adaptor molecule 1 (Iba-1), glial fibrillary acidic protein (GFAP), and neuronal specific nuclear protein (NeuN) at 24 h after ICH. Additionally, triple- labeling immunofluorescence staining was used to co-localize integrin αVβ5 with irisin and Iba-1 at 24 h after ICH.

**Experiment 2**

To determine the effects of irisin treatment on neurobehavior tests and neuroinflammation, neurobehavior tests and brain water content were measured at 24 and 72 h after ICH. For effects of irisin at 24 h after ICH, 30 mice were randomly assigned into five groups: sham, ICH + vehicle (PBS), ICH + irisin (80 μg/kg), ICH + irisin (250 μg/kg), and ICH + irisin (750 μg/kg) (n = 6/group). According to brain water content and neurobehavioral tests, the most effective dosage of irisin at 250 μg/kg was selected and used for neurobehavioral assessment 72 h after ICH, in which an additional 24 mice were randomly divided into four groups: sham+ vehicle (PBS), sham+ irisin (250 μg/kg), ICH + vehicle (PBS), and ICH + irisin (250 μg/kg) (n = 6/group). Mice were randomly assigned to the injection of irisin at a dose of 250 μg/kg intranasally or an equal volume of PBS (vehicle) 30 minutes post-ICH.

**Experiment 3**

To assess the long-term neurobehavioral outcomes after ICH, a total of 40 mice were randomly divided into four groups (n = 10/group): sham+ vehicle (PBS), sham+ irisin (250 μg/kg), ICH + vehicle (PBS), and ICH + irisin (250 μg/kg). On days 7, 14, and 21 post-ICH, the rotarod test was conducted to assess post-stroke motor functions. On days 21-26 post-ICH, the Morris water maze test was performed to evaluate spatial cognitive functions.

**Experiment 4**

To investigate effects of irisin on microglia/macrophage activation, neutrophil infiltration, and neuronal apoptosis at 24 h after ICH, a total of 36 mice were randomized into three groups (n=12/group): sham, ICH + vehicle (PBS) and ICH + irisin (250 μg/kg). Among these mice, 18 mice were randomly divided into three groups (n=6/group) for immunofluorescence staining of Iba-1, IL-1β, and myeloperoxidase (MPO), Fluoro-Jade C staining, and terminal deoxynucleotidyl transferase dUTP nick end labeling (TUNEL) co-staining with neuronal marker (NeuN). Immunofluorescence staining of Iba-1, IL-1β, and MPO was quantified by counting Iba-1, IL-1β, and MPO positive cells in the perihematomal area (20×, averaged from 3 field view/slice, 10 slices/mouse). To quantify the protein levels of Iba-1, IL-1β, MPO, Bax and Bcl-2, an additional 18 mice were randomly divided into three groups for western blot analysis (n = 6/group): sham, ICH + vehicle (PBS), ICH + irisin (250 μg/kg).

**Experiment 5**

To evaluate effects or irisin on microglia/macrophage polarization at 72 h after ICH, 18 mice were randomly assigned into three groups (n = 6/group): sham, ICH + vehicle (PBS), ICH + irisin (250 μg/kg). Immunofluorescence staining (n = 6/group) of Iba-1/CD16 and Iba-1/CD206 was quantified by counting Iba-1/CD16 and Iba-1/CD206 positive cells in the perihematomal area (20×, averaged from 3 field view/slice, 10 slices/mouse). To further assess effects of irisin on microglia/macrophage polarization, 18 mice were randomized into three groups to perform quantitative real-time PCR (q-PCR): sham, ICH + vehicle (PBS), ICH + irisin (250 μg/kg) (n = 6/group).

**Experiment 6**

To elucidate the role of integrin αVβ5 receptor in irisin-mediated neuroinflammation and neuronal apoptosis after ICH, a total of 30 mice were randomly divided into five groups (n = 6/group): sham, ICH + vehicle (PBS), ICH + irisin (250 μg/kg), ICH+ irisin + cilengitide, ICH + irisin + dimethyl sulfoxide (DMSO). Cilengitide, a selective integrin αVβ5 inhibitor was administered intraperitoneally (i.p.) at 2 h before ICH induction followed by the intranasal (i.n.) delivery of irisin (250 μg/kg) at 30 min after ICH. Neurobehavioral tests and western blot analysis were performed at 24 h post-ICH.

**Experiment 7**

To further explore the AMPK signaling pathway in irisin-mediated neuroinflammation after ICH, a total of 30 mice were randomized into five groups (n = 6/group): sham, ICH + vehicle (PBS), ICH + irisin (250 μg/kg), ICH+ irisin + dorsomorphin, ICH + irisin + DMSO. The AMPK inhibitor, dorsomorphin, was administrated

intracerebroventricularly (i.c.v) 30 minutes prior to ICH injury. Neurobehavioral tests and western blot were performed at 24 h after ICH induction.

**Part 2:**

|  | Table S1. Summary of experimental groups and mortality rate in the study. | | | | | | | |
| --- | --- | --- | --- | --- | --- | --- | --- | --- |
|  |  |  |  |  |  |  |  |  |
| **Experimental Groups** | | **Neurological test / BWC** | **IHC**  **TUNEL**  **FJC** | **WB**  **ELISA** | **qPCR** | **Exclusion** | **Mortality (%)** | **Subtotal** |
| **Experimental 1** | |  |  |  |  |  |  |  |
| Sham | |  | 2 | 6 |  | 0 | 0 | 8 |
| ICH (3h, 6h, 12h, 24h, 72h, 7d) | |  | 2 | 36 |  | 1 | 2(4.88%) | 41 |
| **Experimental 2** | |  |  |  |  |  |  |  |
| Sham + Vehicle | | 12 |  |  |  | 0 | 0 | 12 |
| ICH + Vehicle | | 12 |  |  |  | 0 | 1(7.69%) | 13 |
| ICH + Irisin 80 ug/kg  ICH + Irisin 250 ug/kg  ICH + Irisin 750 ug/kg  Sham + Irisin 250 ug/kg | | 6  12  6  6 |  |  |  | 0  0  0  0 | 0  0  0  0 | 6  12  6  6 |
| **Experimental 3** | |  |  |  |  |  |  |  |
| Sham + Vehicle  Sham + Irisin | |  | 10  10 |  |  | 0  0 | 0  0 | 10  10 |
| ICH + Vehicle | |  | 10 |  |  | 1 | 1(8.33%) | 12 |
| ICH + Irisin | |  | 10 |  |  | 0 | 0 | 10 |
| **Experimental 4** | |  |  |  |  |  |  |  |
| Sham | |  | 6 | 6 |  | 0 | 0 | 12 |
| ICH + Vehicle | |  | 6 | 6 |  | 0 | 1(7.69%) | 13 |
| ICH + Irisin | |  | 6 | 6 |  | 0 | 0 | 12 |
| **Experimental 5** | |  |  |  |  |  | | |
| Sham | |  | 6 |  | 6 | 0 | 0 | 12 |
| ICH + Vehicle | |  | 6 |  | 6 | 0 | 1(7.69%) | 13 |
| ICH + Irisin | |  | 6 |  | 6 | 0 | 1(7.69%) | 13 |
| **Experimental 6** | |  |  |  |  |  |  |  |
| Sham | |  |  | 6 |  | 0 | 0 | 6 |
| ICH + Vehicle  ICH + Irisin  ICH + Irisin + Cilengitide  ICH + Irisin + DMSO  **Experimental 7**  Sham  ICH + Vehicle  ICH + Irisin  ICH + Irisin + Dorsomorphin  ICH + Irisin + DMSO | |  |  | 6  6  6  6    6  6  6  6  6 |  | 0  1  0  0    0  0  0  0  0 | 1(14.29%)  0  0  1(14.29%)    0  1(14.29%)  0  0  0 | 7  7  6  7    6  7  6  6  6 |
| **Total** | | 54 | 80 | 120 | 18 | 3 | 10(4.93%) | 285 |
|  | ICH, intracerebral hemorrhage; WB, western blot; IHC, immunohistochemistry; TUNEL, transferase dUTP nick end labeling; FJC, Fluoro-Jade C staining; ELISA, enzyme-linked immunosorbent assay | | | | | | | |

**Part 3: Primers for qPCR**

**Primers (5’-3’)**

GADPH Forward GTGAAGGTCGGTGTGAACGG

GADPH Reserve GTTTCCCGTTGATGACCAG

CD16 Forward TTTGGACACCCAGATGTTTCAG

CD16 Reserve GTCTTCCTTGAGCACCTGGATC

CD32 Forward AATCCTGCCGTTCCTACTGATC

CD32 Reserve GTGTCACCGTGTCTTCCTTGAG

IL-1β Forward CTCCATGAGCTTTGTACAAGG

IL-1β Reserve TGCTGATGTACCAGTTGGGG

iNOS Forward CAAGCACCTTGGAAGAGGAG

iNOS Reserve AAGGCCAAACACAGCATACC

IL-6 Forward ACACATGTTCTCTGGGAAATC

IL-6 Reserve AGTGCATCATCGTTGTTCATA

CD11b Forward CCAAGACGATCTCAGCATCA

CD11b Reserve TTCTGGCTTGCTGAATCCTT

CD206 Forward CAAGGAAGGTTGGCATTTGT

CD206 Reserve CCTTTCAGTCCTTTGCAAGC

Arg1 Forward TCACCTGAGCTTTGATGTCG

Arg1 Reserve CTGAAAGGAGCCCTGTCTTG

CCL22 Forward CTGATGCAGGTCCCTATGGT

CCL22 Reserve GCAGGATTTTGAGGTCCAGA

TGF-β Forward TGCGCTTGCAGAGATTAAAA

TGF-β Reserve CGTCAAAAGACAGCCACTCA

IL-13 Forward CCTGGCTCTTGCTTGCCTT

IL-13 Reserve GGTCTTGTGTGATGTTGCTCA

YM1/2 Forward CAGGGTAATGAGTGGGTTGG

YM1/2 Reserve CACGGCACCTCCTAAATTGT

**Part 4: Supplementary figures**

**Figure S2. Irisin expression in the microglia/macrophage in the sham and ICH mice**


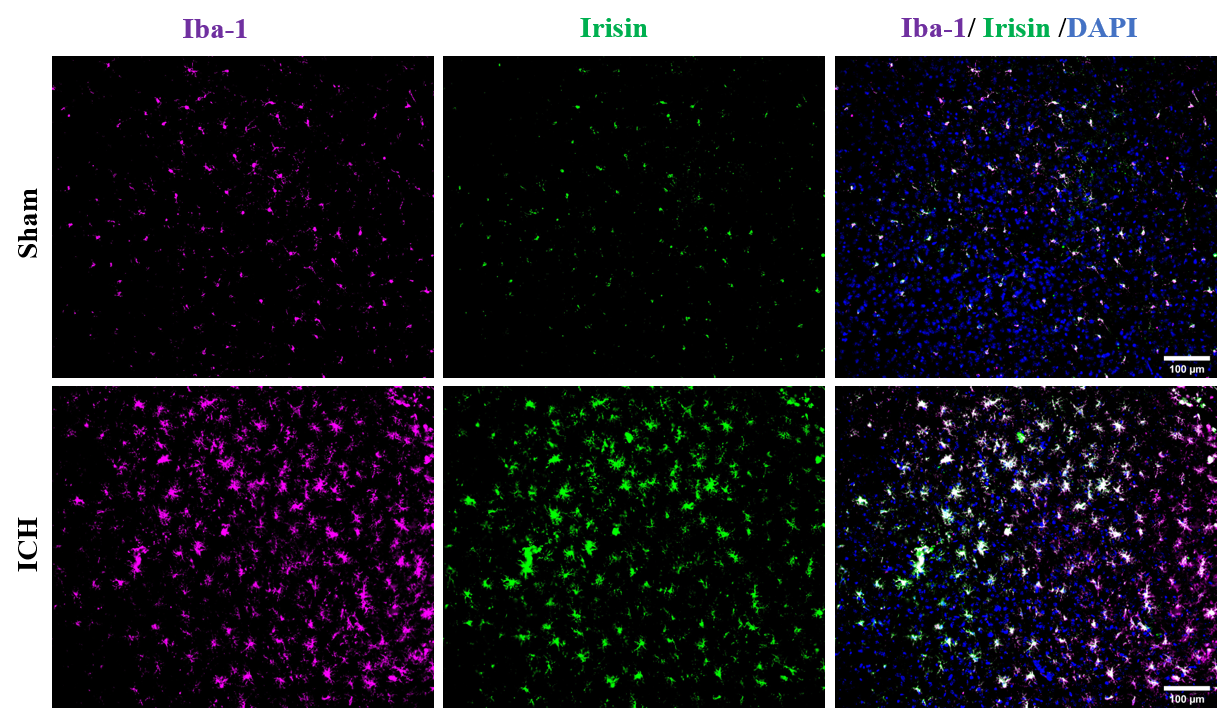


Figure S2. Representative double immunofluorescence staining for Iba1 (magenta) and irisin (green) in sham group and the perihematomal area of ICH (24h) group. Scale bar = 100 μm.

**Figure S3: Time-course expression of irisin between vehicle-treated and irisin-treated ICH mice**


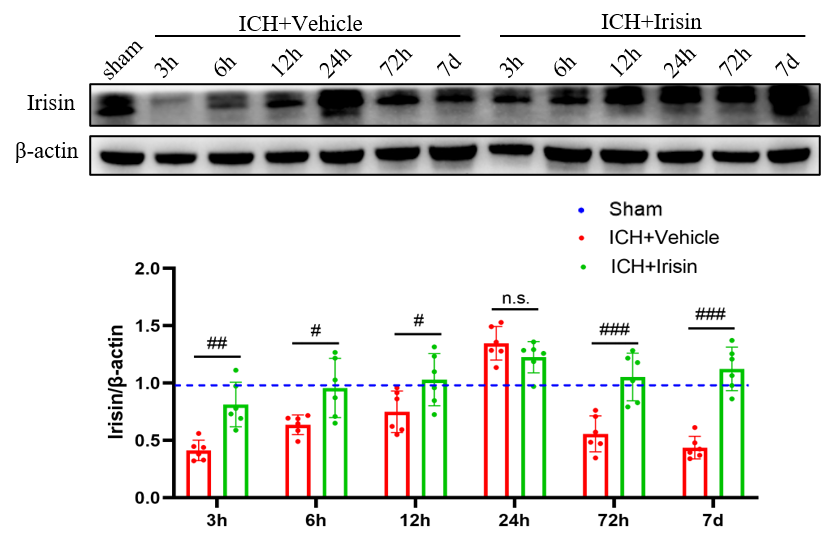


Figure S3. Representative western blot bands and quantitative analyses of time course of irisin expression in the ipsilateral hemisphere after ICH in vehicle and irisin-treated ICH groups. #*p* < 0.05, ##*p* < 0.01, ###*p* < 0.001 vs. ICH + vehicle group, mean ± SD, n=6/group.
